# Mesenteric Panniculitis in a Renal Transplant Patient With Systemic Lupus Erythematosus

**DOI:** 10.7759/cureus.80340

**Published:** 2025-03-10

**Authors:** Elizabeth Geyer-Roberts, Julia Grote, Andrew Banuelos

**Affiliations:** 1 Dr. Kiran C. Patel College of Osteopathic Medicine, Nova Southeastern University, Fort Lauderdale, USA; 2 Internal Medicine, Jackson Memorial Hospital, Miami, USA

**Keywords:** immunosuppressive drugs, lupus erythematosus panniculitis, mesenteric panniculitis, renal transplant surgery, systemic lupus erythematosus

## Abstract

Mesenteric panniculitis (MP) is a disease of unknown etiology, causing fibrosis of the small bowel mesentery. MP is typically diagnosed incidentally with computed tomography imaging but is occasionally associated with surgery, trauma, and cancer. The prevalence of MP varies across the literature; however, it is agreed upon to be a rare diagnosis. This case describes a 34-year-old female with systemic lupus erythematosus (SLE) who presented with abdominal symptoms and was later found to have MP. We postulated that the patient’s SLE flare and adjustment in immunosuppressive medications facilitated the development of MP. This case highlights the need for further research regarding the association of MP and autoimmune conditions such as SLE.

## Introduction

Mesenteric panniculitis (MP) is a disease of chronic inflammation causing fibrosis of the small bowel mesentery [1]. Although the etiology is unknown, MP is typically associated with surgery, trauma, and cancer [1]. There are few reported associations with autoimmune diseases, including Sjögren’s syndrome, systemic lupus erythematosus (SLE), and rheumatoid arthritis [2]. MP is often discovered incidentally as many patients can be asymptomatic; however, many patients can develop non-specific symptoms such as abdominal pain and tenderness [2]. The diagnosis often requires an extensive workup that includes laboratory studies combined with computed tomography (CT) imaging while ruling out other disease processes. CT imaging studies can reveal pathognomonic features such as mass effect, mesenteric fat tissue demonstrating inhomogeneous attenuation, small soft tissue nodules, a halo sign, and a pseudocapsule. The estimated prevalence of MP can vary widely, ranging from 0.6% to 7.8% in different studies [3]. Some studies relied upon the formal radiologist interpretation of the abdominal imaging, whereas others re-evaluated the imaging results directly to measure the prevalence of MP, which causes the large variation in reported prevalence [3].

Although MP has been covered extensively in the existing literature, cases associated with SLE are very rare, with only four published reports currently [4-7]. We present the case of MP diagnosed in a young female with a history of SLE status post-renal transplant complicated by a SLE flare during pregnancy. Furthermore, this case demonstrates how MP can present at various stages of the disease process, as the autoimmune serology levels in our patient indicated well-controlled autoimmune disease overall. This case report aims to bring awareness to broad etiologies of MP and the importance of CT imaging to guide diagnosis in cases with unexplained symptoms.

## Case presentation

A 34-year-old Jamaican female with a past medical history significant for SLE and end-stage renal disease due to lupus nephritis presented to our institution with complaints of intermittent abdominal pain, right-sided groin pain, nausea, and vomiting for multiple weeks. The patient was diagnosed with SLE in 2010 after a long history of non-specific rashes, two seizures, and an episode of unilateral leg swelling that provoked further workup. Her past surgical history was significant for a renal transplant in 2016 for the treatment of lupus nephritis that resulted in the cessation of dialysis and medical management of her underlying conditions. The patient’s medications included tacrolimus, low-dose prednisone, and mycophenolate after the renal transplant. With her current therapy, graft function remained stable and SLE remained quiescent for many years. Mycophenolate, a known teratogen, was replaced with azathioprine while she attempted to become pregnant. She was unsuccessful for the following three years with multiple spontaneous abortions.

On physical examination, the patient was in no acute distress. The abdominal examination revealed mild tenderness to palpation of the lower abdominal quadrants, with normoactive bowel sounds. No distension or gross deformities of the abdomen were noted. Vital signs were unremarkable.

**Figure 1:**
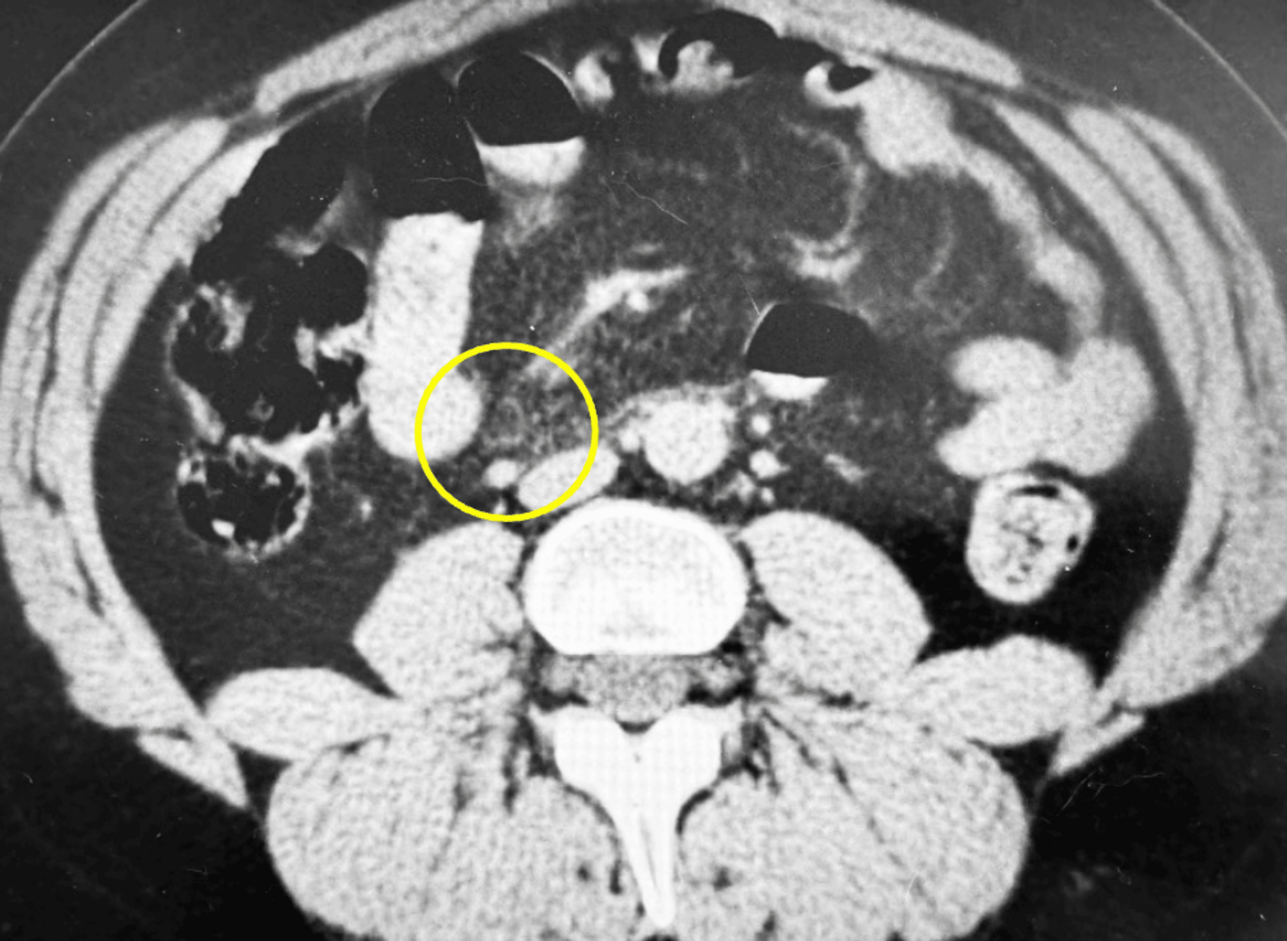
Axial CT image showing mesenteric panniculitis.

The patient’s laboratory results, including her complete blood count, liver function tests, and tacrolimus level, were within normal limits. Complement C3 and erythrocyte sedimentation rate were slightly elevated at 169 mg/dL (normal: 80-160 mg/dL) and 44 mm/hour (normal: <20 mm/hour), respectively. All other laboratory studies were within normal limits. CT of the abdomen and pelvis was performed and revealed MP and a small right-sided inguinal hernia (Figure 1). Treatment of the patient involved a multidisciplinary approach. We resumed her immunosuppressant medications to address and treat the MP and consulted surgical specialists to address the inguinal hernia. After discharge, the patient followed up in the outpatient nephrology clinic and reported resolution of her symptoms.

## Discussion

Cases involving an autoimmune etiology of MP in renal transplant patients have not been explored in the literature. This is a unique case that follows the workup and management of a 34-year-old female who was diagnosed with MP eight years following a renal transplant due to lupus nephritis. At the time of admission and subsequent diagnosis, she was taking azathioprine, which has been used as treatment for other cases of MP [1]. Failure to respond proved that single drug therapy was not enough for this case of MP. The patient was placed on combination therapy of tacrolimus, mycophenolate, and prednisone and reported resolution of symptoms three months later.

The pathophysiology of MP, both in general and in patients with autoimmune conditions, remains poorly understood. Before her MP diagnosis, autoimmune diagnostic serologies in our patient indicated well-controlled autoimmune disease. Regardless of well-controlled SLE, the disease is associated with eliciting hypercoagulable states in affected patients, with thrombosis as one of the most common causes of death [8]. Although MP does not have a definitive cause, mesenteric thrombosis is thought to be a contributing factor. Mesenteric thrombosis creates microthrombi that cause chronic inflammation and associated scarring seen in MP [8]. It is possible that this patient’s hypercoagulability contributed to the development of her MP via mesenteric thrombosis. Antiphospholipid syndrome (APS), which is a hypercoagulable clotting disorder commonly seen in SLE, is a known cause of mesenteric thrombosis. Up to 30-40% of patients with SLE are positive for antiphospholipid antibodies and up to 10% are positive for APS [9,10]. Unfortunately, this patient was not tested for APS at the time of presentation; however, this case acknowledges the importance of APS testing for patients with SLE who present with abdominal symptoms.

The management of MP can vary depending on the severity of illness. Asymptomatic patients with MP do not require treatment, as it is benign [11]. The largest case series to date on MP, involving 92 cases with a median age of 65 years, examined 20 cases that were treated with tamoxifen and a prednisone taper [12]. Overall, 12 of the 20 patients responded to this regimen [12]. There is also evidence suggesting that treatment with colchicine plus prednisone was as effective as therapy involving tamoxifen with prednisone [13]. Biologic therapy has also been used for recalcitrant cases, but currently there are only a few publications documenting this treatment option [11]. Thus, the treatment of MP can involve diverse regimens and must continue to be investigated.

Lastly, although SLE is associated with a variety of gastrointestinal disorders, this case report also aims to bring awareness to the importance of thorough imaging studies in lupus patients, even when symptomatology is mild or intermittent or when diagnostic serologies are inconclusive. Although biopsy is the definitive means of diagnosing MP, CT imaging is more commonly used as MP can demonstrate pathognomonic radiologic features such as a fat halo sign, pseudocapsule, or a greasy ring signal among others [1].

## Conclusions

In conclusion, we suspected that the adjustment in immunosuppressive regimen during pregnancy may have precipitated a lupus flare with the additional development of MP. We expected that the reconstitution of the original combination maintenance medication profile of tacrolimus, mycophenolate, and prednisone would be successful in treating her MP.
